# Relationship of Personal Hygiene with Nutrition and Morbidity Profile: A Study Among Primary School Children in South Kolkata

**DOI:** 10.4103/0970-0218.66894

**Published:** 2010-04

**Authors:** Soumya Deb, Sinjita Dutta, Aparajita Dasgupta, Raghunath Misra

**Affiliations:** Department of Community Medicine, IPGMER, SSKM Medical College and Hospital, Kolkata, India; 1 Department of Preventive and Social Medicine (PSM), All India Institute of Hygiene and Public Health, Kolkata, India

**Keywords:** Morbidity, personal hygiene, school children

## Abstract

**Background:**

Majority of the health problems affecting school children are preventable by promotion of hygienic practices through proper health education by the teachers, who are the first contacts.

**Objectives:**

The study was undertaken to find out the status of nutrition and personal hygiene among primary school children and their association with their varied morbidity profiles.

**Materials and Methods:**

A descriptive, observational, cross-sectional study was conducted in a primary school situated in the largest slum of Kolkata.

**Results:**

The participants included 103 boys and 81 girls, with a mean age of 6.2 years. The mean personal hygiene score of the girls (4.15 ± 0.98) was significantly higher than that of boys (3.2 ± 1.4) [*P*<0.05]. Most of the boys (54.37%) and girls (74.07%) were normally nourished as per the CDC growth chart. Over 70% of the children were suffering from one or more morbidities, the most common morbidity in both the sexes being pallor, followed by worm infestation. Personal hygiene scores were significantly higher (*P*<0.05) among those children who were normally nourished as well as those who did not suffer from any morbidity in the last 15 days.

**Conclusions:**

Care should be taken to improve the pitiable state of personal hygiene and poor sanitary practices of these school children through coordinated and concerted health education measures by teachers as well as parents.

## Introduction

The importance of school health has been acknowledged across countries since the beginning of 20^th^ century. In several developed countries, school health programs have evolved during the post-2^nd^ World War period and addressed nutritional and physical-fitness aspects. This was in response to poor nutritional status among lower middle class and working class children. School health services have tended to focus on nutritional support and clinical assessment. These inputs are absolutely necessary but so is the need to assess the state of personal hygiene, which is directly or indirectly related to the above-mentioned factors, especially in a developing country like India.(1)

Schools are sacred because they provide an environment for learning skills, and for development of intelligence that can be utilized by students to achieve their goals in life. It is also observed that “to learn effectively children need good health.” Health is a key factor in school entry, as well as continued participation and attainment in school.(2) School is the place where health education regarding important aspects of hygiene, environment and sanitation, as well as social customs, is being imparted. The teacher is the guardian of the child in school and plays a pivotal role in the whole process of primordial prevention.

There are about 6.3 lakh schools in India, both primary and upper primary, with 128.3 million children in primary schools and about 50 million in upper primary schools.(1) But it is also a fact that only 8% of the schools have sanitation facilities in school premises, only 44% have water supply facilities, 19% have urinals and 8% have lavatory facilities. For girls, barely 19% have separate urinals; and 4% separate lavatory facility.(3) The consequences of the given situation are obvious. Diarrhoea takes a heavy toll. Typhoid, dysentery, gastroenteritis, hepatitis-A, intestinal worms and malaria continue to kill, debilitate and contribute to the high rates of malnutrition among young children in the country. Intestinal parasites are among the most common infections in school-age children in developing countries. As a result of this morbidity, they are at risk of detrimental effects like poor cognitive performance and physical growth.(4) Majority of these diseases are largely preventable by promotion of hygienic practices among school children through proper health education by the teachers, who are the first contacts. Therefore, coordinated and regular activities pertaining to health and hygiene at school are needed, especially health check-up and de-worming, for better and healthy school environment.(5,6)

The present study was undertaken among primary school children in a school of south Kolkata, with the following objectives:

1. To find out the status of personal hygiene among the primary school children.
2. To find out the nutritional status and morbidity profile of the study population.
3. To elicit the relationship of personal hygiene with its morbidity pattern.

## Materials and Methods

A health check-up program for primary school children studying in class I and class II was organized by the researchers over a period of 2 months (July and August 2008) after taking necessary permission from the Principal at the Chetla School. The school is a primary co-education school comprising of classes I to IV, where children mainly from neighboring slum locality study. Because of the ongoing exams, students of class III and IV were excluded. The age of the study participants ranged from 5 to 10 years. There were two sections each in class I and class II. Students of all the sections were taken for check-up during the study period. A total of 204 children were enrolled from the four sections, of which there were 20 absentees. Thus a total of 184 students participated in the study.

The tools included pre-tested and pre-designed questionnaire, weighing machine and measuring tape. General and systemic examinations of the students were conducted. The children were also interviewed about personal hygiene practices using the Global School Health Survey Questionnaire. Any morbidity suffered by the students during the last 15 days was recorded, like Diarrhoea, worm infestation or cough and cold. Data thus collected were analyzed using suitable statistical tests with the help of Microsoft Excel 2007 and EpiInfo version 3.2.

## Results and Analysis

The study included 103 boys and 81 girls. Majority (76.9%) of the participants were aged between 6 and 7 years, with a mean age of 6.2 years. As shown in Table 1, the status of personal hygiene was considered either favorable (score of 1) or unfavorable (score of 0). Uniformity of assigning scores was maintained throughout the study because the decision as to whether the state of each variable of personal hygiene was favorable or not was taken jointly by all the researchers. Regarding habits/practices related to personal hygiene, a score=0 corresponded to “never practicing”; a score=1, to “sometimes practicing”; and a score=2, to “practicing most of the times.” Therefore, overall, the maximum and minimum possible scores were 13 and 0, respectively. A closer look reveals that the status of personal hygiene among girls was better as compared to boys when it came to clean and trimmed nails (77.8% *vs*. 50.5%, *P*<0.05) and clean hands and skin (92.6% *vs*. 68.9%, *P*<0.05). With regard to clean uniform and cleanliness of tooth and tongue, the results were more or less the same for both sexes. Clean/combed hair was found more in boys as compared to girls (92.23% *vs*. 85.19%). Girls fared better than boys regarding regular use of soap for hand washing at school (11.1% *vs*. 5.8%), regular hand washing after visiting toilet (92.6% *vs*. 73.8%, *P*<0.05), as well as regular use of soap for hand washing at home (33.3% *vs*. 27.2%). However, more boys used toothpaste and toothbrush regularly (67%) as compared to girls (55.6%). The rest unfortunately followed unhygienic practices like using fingers, toothpowder, etc. The overall mean score of the study population was 3.4 ± 1.3. The mean score for girls (4.15 ± 0.98) was significantly higher than that for boys (3.2 ± 1.4) [*P*<0.05]. Majority (51.5%) of the study participants scored in the range of 3-4.

**Table 1:** Distribution of the study population according to status of personal hygiene (*n*= 184)

State of personal hygiene	Unfavorable state (Score 0)		Favorable state (Score 1)		Test of significance (χ^2^, *P* value, df)		
	M(*n*=103)	F(*n*=81)	M(*n*=103)	F(*n*=81)			
Hair clean/combed	8 (7.77)	12 (14.81)	95 (92.23)	69 (85.19)	1.65, >0.05, 1		
Nails trimmed/clean	51 (49.51)	18 (22.22)	52 (50.49)	63 (77.78)	13.27, <0.05, 1		
Uniform clean	17 (16.5)	15 (18.52)	86 (83.5)	66 (81.48)	0.03, >0.05, 1		
Clean hands, feet and skin	32 (31.07)	6 (7.41)	71 (68.93)	75 (92.59)	14.08, <0.05, 1		
Clean oral cavity	29 (28.16)	24 (29.63)	74 (71.84)	57 (70.37)	0.05, >0.05, 1		

**Practices related to personal hygiene**	**Never practiced (SCORE 0)**		**Sometimes practiced (Score 1)**		**Practiced most of the times (Score 2)**		**Test of significance (χ^2^, *P* value, df)**
	**M(n=103)**	**F(n=81)**	**M(n=103)**	**F(n=81)**	**M(n=103)**	**F(n=81)**	

Use of soap for hand washing at school	61 (59.2)	30 (37.03)	36 (34.9)	42 (51.85)	6 (5.8)	9 (11.11)	1.69, >0.05, 1
Hand washing after toilet	8 (7.6)	-	19 (18.5)	6 (7.4)	76 (73.8)	75 (92.6)	9.66, <0.05, 1
Use of soap for hand washing at home	30 (29.1)	15 (18.5)	45 (43.7)	39 (48.2)	28 (27.2)	27 (33.3)	0.55, >0.05, 1
Use of toothpaste with toothbrush	25 (24.3)	30 (37.0)	9 (8.7)	6 (7.4)	69 (67.0)	45 (55.6)	2.05, >0.05, 1
Total score obtained	Males No. (%)		Females No. (%)		Total No. (%)		
Poor (1-2)	27 (26.2)		9 (11.11)		36 (19.57)		
Average (3-4)	56 (54.4)		36 (44.44)		92 (50)		
Good (5-6)	20 (19.4)		36 (44.44)		56 (30.43)		
Total	103 (100)		81 (100)		184 (100)		
Mean±SD	3.12±1.4		4.15±0.98		3.4±1.3		
Range	1-6		2-5		1-6		
Unpaired *t* critical two-tailed value=1.973, *P*<0.05, df=182

M=males, F=females

Running water was used for hand washing by 59.23% of the children at home and 82.3% at school. Other sources of water for washing hands at home included a common dish of water shared by several persons or a dish of water used only by the child. Almost 18% of the children never washed their hands before eating while at school.

The girls had better hand washing practices than the boys before eating at home (70.4% *vs*. 56.3%), as well as at school (92.6% *vs*. 79.6%).

It was found that 76% of the boys and 74% of the girls were suffering from one or more morbidities. History was elicited for the last 15 days to avoid recall bias. This was followed by thorough clinical examination of each student. For boys, the most common morbidity was clinically detected pallor (55.34%), followed by undernutrition (40.78%) and worm infestation (39.81%). The most common morbidity for girls was, again, clinically detected pallor (51.85%), followed by caries in teeth (33.34%) and worm infestation (29.63%).

Table 2 shows the distribution of the study population according to its nutritional status as per CDC 2000 growth chart guidelines.(7) It was observed that the boys were more undernourished than the girls (40.78% *vs*. 25.93%), and this difference was statistically significant (*P*<0.05).

**Table 2:** Distribution of study population according to nutritional status* (*n*= 184)

Nutritional status	Males No. (%) *n*=103	Females No. (%) *n*=81
Underweight (BMI<5^th^ percentile)	42 (40.78)	21 (25.93)
Normal nutritional status (BMI 5^th^ - 84^th^ percentile)	56 (54.37)	60 (74.07)
At risk of overweight (BMI 85^th^ - 95^th^ percentile)	2 (1.94)	-
Overweight (BMI>95^th^ percentile)	3 (2.91)	-
Total (%)	103 (100)	81 (100)
Chi square=4.44, *P*<0.05, df=1		

* As per CDC 2000 growth chart for age 2-20 years

Table 3 shows that by and large the study population that did not suffer from any morbidity over the last 15 days had significantly higher personal hygiene scores (*P*<0.05) as compared to those that suffered from one or more morbidities. Higher scores, viz., 5-6, were secured by 40.6% of the study participants without any morbidity, whereas similar scores were secured by only 19.4% of those who suffered from one or more morbidities. Similarly only 3.1% of those without any morbidity secured low personal hygiene scores, viz., 1-2, as compared to 29.6% in the group with morbidity.

**Table 3:** Relationship of personal hygiene score of the study population with its morbidity profile and nutritional status

Score	Morbidity(*n*=184)		Total	Nutritional status(*n*=184)		Total
	Present	Absent		Underwt	Normal/overwt	
1-2 (Poor)	41 (29.59)	2 (3.13)	43 (39)	35 (51.0)	8 (6.2)	43 (39)		
3-4 (Average)	70 (51.02)	25 (56.25)	95 (88.4)	21 (30.6)	74 (65.4)	95 (88.4)
5-6 (Good)	27 (19.39)	19 (40.62)	46 (41.6)	7 (18.4)	39 (28.4)	46 (41.6)
Total	138 (100)	46 (100)	184 (100)	63 (100)	121 (100)	184 (100)
Chi sq. value=16.11, *P*<0.05, df=2				Chi sq. value=56.07, *P*<0.05, df=2		

Figures in parentheses indicate percentages.

In general, the study populations that were normally nourished/overweight had significantly higher personal hygiene scores (*P*<0.05) as compared to those that were undernourished. Higher scores, viz., 5-6, were secured by 28.4% of the normally nourished/overweight study participants, whereas similar scores were obtained by only 18.4% of those who were undernourished. Lower scores, viz., 1-2, were secured predominantly (51%) by the undernourished group as compared to those who were normally nourished/overweight (6.2%).

## Discussion

A similar study(8) carried out in a school in Wardha district showed the following results. In the Wardha study, 27.6% of the students had clean/combed hair as compared to 92.23% boys and 85.19% girls in the present study, while 29.7% of the students had clean/cut nails as opposed to 77.8% girls and 50.5% boys in the present study. Over 80% of the children irrespective of sex wore clean uniform in the present study as compared to 42.8% of the children in the Wardha study. The percentage of children with good oral hygiene came out to be about 70% in both sexes in the present study as against 33.8% in the Wardha study. Clearly, the findings of the present study were better. May be, the difference in place of study was an important factor, because the present study was conducted in an urban school catering to the largest slum population of Kolkata, while the Wardha study was conducted among tribal primary school children. The most common morbidities among the children in the Wardha study were Diarrhoea, fever, upper respiratory tract infections (RTIs) (56.6%), followed by head lice (42.8%), scabies (36.6%), multiple boils (8.9%) and dental carries (8.3%). However, the present study revealed that the commonest morbidity among boys as well as girls was clinical pallor. History of worm infestation was elicited in 28.9% of the children in the Wardha study as compared to 39.81% of the boys and 29.63% of the girls in the present study.

Another study carried out among school-going (aged 6-14 years) children in Dhotra (Kasar) in Wardha district of central India revealed the prevalence of intestinal parasites to be 17.8%,(9) which was lower than that reported in the present study.

## Conclusion and Recommendation

In India, school health services, including health education, clinical assessment and monitoring of nutritional status, are provided by the primary health centres in rural areas. The same in the urban areas are provided by the respective municipalities. These services in the place of the present study are provided by the Urban Health Centre, Chetla, under the auspices of All India Institute of Hygiene and Public Health. The present study revealed the poor state of personal hygiene among primary school children in the study area. It was observed that the overall status of personal hygiene was better among girls as compared to boys, although boys fared better than girls in some aspects, like clean/ combed hair and regular use of toothpaste. Morbidity and undernutrition were significantly higher in the group with poor personal hygiene score. Hence care should be taken to improve the status of personal hygiene of these school children through coordinated primordial and primary preventive measures like health education. The researchers have imparted health education to these students as well as teachers of the school following the study and plan to organize a series of concerted health education camps for these school children in the months to come. But the onus also lies on teachers and parents. In this aspect, not only parents but also school teachers need to be trained adequately. Besides, there should be monthly parent-teacher meetings so that teachers could give their feedback to parents and vice versa. Unfortunately such meetings are few and far in between. Simple measures like improvement of personal hygiene and following safe, hygienic practices by these children can go a long way in reducing morbidities and thus break the vicious cycle of infection and malnutrition.
